# Supplementary material for: Cord Blood Adipocytokines and Body Composition in Early Childhood: A Systematic Review and Meta-Analysis
Source: Int J Environ Res Public Health. 2021 Feb 16;18(4):1897. doi: 10.3390/ijerph18041897 (PMC7920289; doi:10.3390/ijerph18041897)
Supplement: Supplementary file 1 [file ijerph-18-01897-s001.pdf]

International Journal of
Environmental Research and Public Health

MDPI

NIH National Heart, Lung, and Blood Institute

Quality Assessment Tool for Observational Cohort and Cross-Sectional Studies

Criteria	Yes	No	Other (CD, NR, NA)*
1. Was the research question or objective in this paper clearly stated?			
2. Was the study population clearly specified and defined?			
3. Was the participation rate of eligible persons at least 50%?			
4. Were all the subjects selected or recruited from the same or similar populations (including the same time period)? Were inclusion and exclusion criteria for being in the study prespecified and applied uniformly to all participants?			
5. Was a sample size justification, power description, or variance and effect estimates provided?			
6. For the analyses in this paper, were the exposure(s) of interest measured prior to the outcome(s) being measured?			
7. Was the timeframe sufficient so that one could reasonably expect to see an association between exposure and outcome if it existed?			
8. For exposures that can vary in amount or level, did the study examine different levels of the exposure as related to the outcome (e.g., categories of exposure, or exposure measured as continuous variable)?			
9. Were the exposure measures (independent variables) clearly defined, valid, reliable, and implemented consistently across all study participants?			
10. Was the exposure(s) assessed more than once over time?			
11. Were the outcome measures (dependent variables) clearly defined, valid, reliable, and implemented consistently across all study participants?			
12. Were the outcome assessors blinded to the exposure status of participants?			
13. Was loss to follow-up after baseline 20% or less?			
14. Were key potential confounding variables measured and adjusted statistically for their impact on the relationship between exposure(s) and outcome(s)?			

Quality Rating (Good, Fair, or Poor) (see guidance)
Rater #1 initials:
Rater #2 initials:
Additional Comments (If POOR, please state why):

*CD, cannot determine; NA, not applicable; NR, not reported

Guidance for Assessing the Quality of Observational Cohort and Cross-Sectional Studies

The guidance document below is organized by question number from the tool for quality assessment of observational cohort and cross-sectional studies.

Question 1. Research question

Did the authors describe their goal in conducting this research? Is it easy to understand what they were looking to find? This issue is important for any scientific paper of any type. Higher quality scientific research explicitly defines a research question.

Questions 2 and 3. Study population

Did the authors describe the group of people from which the study participants were selected or recruited, using demographics, location, and time period? If you were to conduct this study again, would you know who to recruit, from where, and from what time period? Is the cohort population free of the outcomes of interest at the time they were recruited?

An example would be men over 40 years old with type 2 diabetes who began seeking medical care at Phoenix Good Samaritan Hospital between January 1, 1990 and December 31, 1994. In this example, the population is clearly described as: (1) who (men over 40 years old with type 2 diabetes); (2) where (Phoenix Good Samaritan Hospital); and (3) when (between January 1, 1990 and December 31, 1994). Another example is women ages 34 to 59 years of age in 1980 who were in the nursing profession and had no known coronary disease, stroke, cancer, hypercholesterolemia, or diabetes, and were recruited from the 11 most populous States, with contact information obtained from State nursing boards.

In cohort studies, it is crucial that the population at baseline is free of the outcome of interest. For example, the nurses' population above would be an appropriate group in which to study incident coronary disease. This information is usually found either in descriptions of population recruitment, definitions of variables, or inclusion/exclusion criteria.

You may need to look at prior papers on methods in order to make the assessment for this question. Those papers are usually in the reference list.

If fewer than 50% of eligible persons participated in the study, then there is concern that the study population does not adequately represent the target population. This increases the risk of bias.

Question 4. Groups recruited from the same population and uniform eligibility criteria

Were the inclusion and exclusion criteria developed prior to recruitment or selection of the study population? Were the same underlying criteria used for all of the subjects involved? This issue is related to the description of the study population, above, and you may find the information for both of these questions in the same section of the paper.

Figure S1. NIH quality assessment tool (available at: https://www.nhlbi.nih.gov/health-topics/study-quality-assessment-tools). [70]

Table S1. Studies were assessed using the NIH quality assessment tool for observational cohort and cross sectional studies.

Study	Was the research question or objective in this paper clearly stated?	Was the study population clearly specified and defined?	Was the participation rate of eligible persons at least 50%?	Were all the subjects selected or recruited from the same or similar populations?	Was a sample size justification, power description, or variance and effect estimates provided?	For the analyses in this paper, were the exposure(s) of interest measured prior to the outcome(s) being measured?	Was the timeframe sufficient so that one could reasonably expect to see an association between exposure and outcome if it existed?	For exposures that can vary in amount or level, did the study examine different levels of the exposure?	Were the exposure measures (independent variables) clearly defined, valid, reliable, and implemented consistently across all study participants?	Was the exposure(s) assessed more than once over time?	Were the outcome measures (dependent variables) clearly defined, valid, reliable, and implemented? consistently across all study participants?	Were the outcome assessors blinded to the exposure status of participants?	Was loss to follow-up after baseline 20% or less?	Were key potential confounding variables measured and adjusted statistically for their impact on the relationship? between exposure(s) and outcome(s)?	Summary Quality
Meyer, 2018 [50]	✔	✔	NR	✔	✗	✔	✔	✔	✔	NA	✔	NR	✗	✔	i
Meyer, 2017 [51]	✔	✔	✔	✔	✗	✔	✔	✔	✔	✔	✔	NR	✗	✔	i i
Schneider, 2017 [49]	✔	✔	✔	✔	✗	✔	✔	✔	✔	NA	✔	NR	✔	✗	i
Kadakia, 2016 [37]	✔	✔	✔	✔	✔	✔	✔	✔	✔	✗	✔	NR	NA	✔	i i
Karakosta, 2016 [43]	✔	✔	✗	✔	✗	✔	✔	✔	✔	✔	✔	NR	✔	✔	i i
Chaoimh, 2016 [36]	✔	✔	✔	✔	✗	✔	✔	✔	✔	✔	✔	NR	✗	✔	i i
Donnelly, 2015 [29]	✔	✔	✔	✔	✗	✔	✔	NA	✔	NA	✔	NR	NA	✔	i
Teague, 2015 [32]	✔	✔	✔	✔	✗	✔	✔	NA	✔	NA	✔	NR	✗	✔	i
Josefson, 2014 [33]	✔	✔	✔	✔	✔	✔	✔	✔	✔	✗	✔	✗	NA	✔	i i
Brunner, 2014 [39]	✔	✔	✔	✔	✗	✔	✔	✔	✔	✔	✔	✗	✔	✔	i i
Boeke, 2013 [45]	✔	✔	✗	✔	✗	✔	✔	✔	✔	✔	✔	NR	✔	✔	i i
Simon-Muela, 2013 [40]	✔	✔	✗	✔	✗	✔	✔	NA	✔	✔	✔	NR	NR	✔	i
Basu, 2009 [41]	✔	✗	NA	NR	✗	✔	✔	✔	✔	NA	✔	NR	NA	✔	i
Mantzoros, 2009 [44]	✔	✔	✗	✔	✗	✔	✔	✔	✔	✔	✔	NR	✔	✔	i i
Inami, 2007 [42]	✔	✔	✔	✔	✗	✔	✔	NA	✔	✔	✔	NR	✔	✗	i
Valuniene, 2007 [35]	✔	✗	✔	✔	✗	✔	✔	NA	✔	NA	✔	NR	✗	✔	i

Martinez, 2005 [30]	✔	✗	NA	✔	✗	✔	✔	NA	✔	NA	✔	✗	NA	✗	i
Javaid, 2005 [31]	✔	✔	✔	✔	✗	✔	✔	NA	✔	✔	✔	✔	NA	✔	i i
Tsai, 2004 [34]	✔	✔	✔	✔	✗	✔	NA	✗	✔	NA	✔	NR	NA	✔	i
Lindsay, 2003 [52]	✔	✔	✗	✔	✗	✔	✔	NA	✔	NA	✔	NR	NA	✔	i
Geary, 1999 [53]	✔	✔	✔	✔	✗	✔	✔	NA	✔	NA	✔	NR	NA	✔	i
Clapp, 1998 [38]	✔	✗	NR	✔	✗	✔	✔	NA	✔	NA	✔	NR	NA	✔	i

Quality was rated as 0 for poor (0–4 out of 14 questions), i for fair (5–10 out of 14 questions), or ii for good (11–14 out of 14 questions); NA: not applicable, NR: not reported.
